# Data Cleaning: Detecting, Diagnosing, and Editing Data Abnormalities

**DOI:** 10.1371/journal.pmed.0020267

**Published:** 2005-09-06

**Authors:** Jan Van den Broeck, Solveig Argeseanu Cunningham, Roger Eeckels, Kobus Herbst

## Abstract

In this policy forum the authors argue that data cleaning is an essential part of the research process, and should be incorporated into study design.

In clinical epidemiological research, errors occur in spite of careful study design, conduct, and implementation of error-prevention strategies. Data cleaning intends to identify and correct these errors or at least to minimize their impact on study results. Little guidance is currently available in the peer-reviewed literature on how to set up and carry out cleaning efforts in an efficient and ethical way. With the growing importance of Good Clinical Practice guidelines and regulations, data cleaning and other aspects of data handling will emerge from being mainly gray-literature subjects to being the focus of comparative methodological studies and process evaluations. We present a brief summary of the scattered information, integrated into a conceptual framework aimed at assisting investigators with planning and implementation. We recommend that scientific reports describe data-cleaning methods, error types and rates, error deletion and correction rates, and differences in outcome with and without remaining outliers.

## The History of Data Cleaning

With Good Clinical Practice guidelines being adopted and regulated in more and more countries, some important shifts in clinical epidemiological research practice can be expected. One of the expected developments is an increased emphasis on standardization, documentation, and reporting of data handling and data quality. Indeed, in scientific tradition, especially in academia, study validity has been discussed predominantly with regard to study design, general protocol compliance, and the integrity and experience of the investigator. Data handling, although having an equal potential to affect the quality of study results, has received proportionally less attention. As a result, even though the importance of data-handling procedures is being underlined in good clinical practice and data management guidelines [1–3], there are important gaps in knowledge about optimal data-handling methodologies and standards of data quality. The Society for Clinical Data Management, in their guidelines for good clinical data management practices, states: “Regulations and guidelines do not address minimum acceptable data quality levels for clinical trial data. In fact, there is limited published research investigating the distribution or characteristics of clinical trial data errors. Even less published information exists on methods of quantifying data quality” [4].

Data cleaning is emblematic of the historical lower status of data quality issues and has long been viewed as a suspect activity, bordering on data manipulation. Armitage and Berry [5] almost apologized for inserting a short chapter on data editing in their standard textbook on statistics in medical research. Nowadays, whenever discussing data cleaning, it is still felt to be appropriate to start by saying that data cleaning can never be a cure for poor study design or study conduct. Concerns about where to draw the line between data manipulation and responsible data editing are legitimate. Yet all studies, no matter how well designed and implemented, have to deal with errors from various sources and their effects on study results. This problem occurs as much to experimental as to observational research and clinical trials [6,7]. Statistical societies recommend that description of data cleaning be a standard part of reporting statistical methods [8]. Exactly what to report and under what circumstances remains mostly unanswered. In practice, it is rare to find any statements about data-cleaning methods or error rates in medical publications.

Although certain aspects of data cleaning such as statistical outlier detection and handling of missing data have received separate attention [9–18], the data-cleaning process, as a whole, with all its conceptual, organizational, logistical, managerial, and statistical-epidemiological aspects, has not been described or studied comprehensively. In statistical textbooks and non-peer-reviewed literature, there is scattered information, which we summarize in this paper, using the concepts and definitions shown in Box 1.

**Box 1. Terms Related to Data Cleaning**

**Data cleaning:** Process of detecting, diagnosing, and editing faulty data.

**Data editing:** Changing the value of data shown to be incorrect.

**Data flow:** Passage of recorded information through successive information carriers.

**Inlier:** Data value falling within the expected range.

**Outlier:** Data value falling outside the expected range.

**Robust estimation:** Estimation of statistical parameters, using methods that are less sensitive to the effect of outliers than more conventional methods.

The complete process of quality assurance in research studies includes error prevention, data monitoring, data cleaning, and documentation. There are proposed models that describe total quality assurance as an integrated process [19]. However, we concentrate here on data cleaning and, as a second aim of the paper, separately describe a framework for this process. Our focus is primarily on medical research and on practical relevance for the medical investigator.

## Data Cleaning as a Process

Data cleaning deals with data problems once they have occurred. Error-prevention strategies can reduce many problems but cannot eliminate them. We present data cleaning as a three-stage process, involving repeated cycles of screening, diagnosing, and editing of suspected data abnormalities. Figure 1 shows these three steps, which can be initiated at three different stages of a study. Many data errors are detected incidentally during study activities other than data cleaning. However, it is more efficient to detect errors by actively searching for them in a planned way. It is not always immediately clear whether a data point is erroneous. Many times, what is detected is a suspected data point or pattern that needs careful examination. Similarly, missing values require further examination. Missing values may be due to interruptions of the data flow or the unavailability of the target information. Hence, predefined rules for dealing with errors and true missing and extreme values are part of good practice. One can screen for suspect features in survey questionnaires, computer databases, or analysis datasets. In small studies, with the investigator closely involved at all stages, there may be little or no distinction between a database and an analysis dataset.

**Figure 1:**
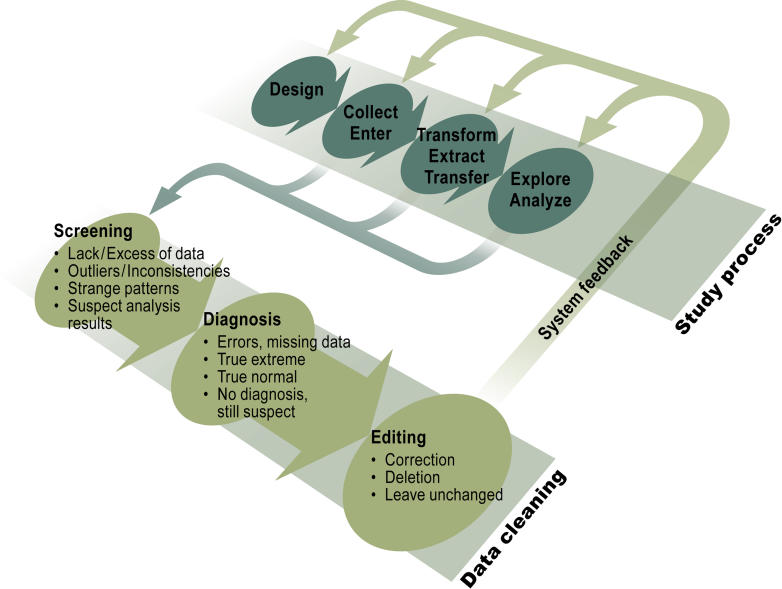
A Data-Cleaning Framework (Illustration: Giovanni Maki)

The diagnostic and treatment phases of data cleaning require insight into the sources and types of errors at all stages of the study, during as well as after measurement. The concept of data flow is crucial in this respect. After measurement, research data undergo repeated steps of being entered into information carriers, extracted, transferred to other carriers, edited, selected, transformed, summarized, and presented. It is important to realize that errors can occur at any stage of the data flow, including during data cleaning itself. Table 1 illustrates some of the sources and types of errors possible in a large questionnaire survey. Most problems are due to human error.

**Table 1:**
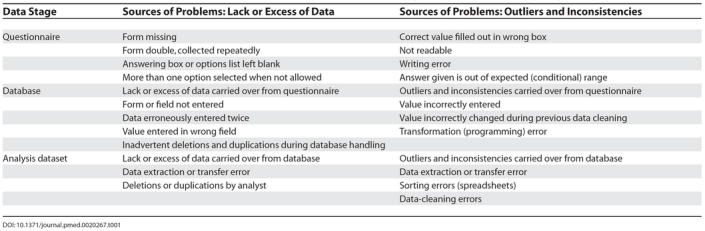
Issues to Be Considered during Data Collection, Management, and Analysis of a Questionnaire Study

Inaccuracy of a single measurement and data point may be acceptable, and related to the inherent technical error of the measurement instrument. Hence, data cleaning should focus on those errors that are beyond small technical variations and that constitute a major shift within or beyond the population distribution. In turn, data cleaning must be based on knowledge of technical errors and expected ranges of normal values.

Some errors deserve priority, but which ones are most important is highly study-specific. In most clinical epidemiological studies, errors that need to be cleaned, at all costs, include missing sex, sex misspecification, birth date or examination date errors, duplications or merging of records, and biologically impossible results. For example, in nutrition studies, date errors lead to age errors, which in turn lead to errors in weight-for-age scoring and, further, to misclassification of subjects as under- or overweight.

Errors of sex and date are particularly important because they contaminate derived variables. Prioritization is essential if the study is under time pressures or if resources for data cleaning are limited.

## Screening Phase

When screening data, it is convenient to distinguish four basic types of oddities: lack or excess of data; outliers, including inconsistencies; strange patterns in (joint) distributions; and unexpected analysis results and other types of inferences and abstractions (Table 1). Screening methods need not only be statistical. Many outliers are detected by perceived nonconformity with prior expectations, based on the investigator's experience, pilot studies, evidence in the literature, or common sense. Detection may even happen during article review or after publication.

What can be done to make screening objective and systematic? To allow the researcher to understand the data better, it should be examined with simple descriptive tools. Standard statistical packages or even spreadsheets make this easy to do [20,21]. For identifying suspect data, one can first predefine expectations about normal ranges, distribution shapes, and strength of relationships [22]. Second, the application of these criteria can be planned beforehand, to be carried out during or shortly after data collection, during data entry, and regularly thereafter. Third, comparison of the data with the screening criteria can be partly automated and lead to flagging of dubious data, patterns, or results.

A special problem is that of erroneous inliers, i.e., data points generated by error but falling within the expected range. Erroneous inliers will often escape detection. Sometimes, inliers are discovered to be suspect if viewed in relation to other variables, using scatter plots, regression analysis, or consistency checks [23]. One can also identify some by examining the history of each data point or by remeasurement, but such examination is rarely feasible. Instead, one can examine and/or remeasure a sample of inliers to estimate an error rate [24]. Useful screening methods are listed in Box 2.

**Box 2. Screening Methods**

- Checking of questionnaires using fixed algorithms.
- Validated data entry and double data entry.
- Browsing of data tables after sorting.
- Printouts of variables not passing range checks and of records not passing consistency checks.
- Graphical exploration of distributions: box plots, histograms, and scatter plots.
- Plots of repeated measurements on the same individual, e.g., growth curves.
- Frequency distributions and cross-tabulations.
- Summary statistics.
- Statistical outlier detection.

## Diagnostic Phase

In this phase, the purpose is to clarify the true nature of the worrisome data points, patterns, and statistics. Possible diagnoses for each data point are as follows: erroneous, true extreme, true normal (i.e, the prior expectation was incorrect), or idiopathic (i.e., no explanation found, but still suspect). Some data points are clearly logically or biologically impossible. Hence, one may predefine not only screening cutoffs as described above (soft cutoffs), but also cutoffs for immediate diagnosis of error (hard cutoffs) [10]. Figure 2 illustrates this method. Sometimes, suspected errors will fall in between the soft and hard cutoffs, and diagnosis will be less straightforward. In these cases, it is necessary to apply a combination of diagnostic procedures.

**Figure 2:**
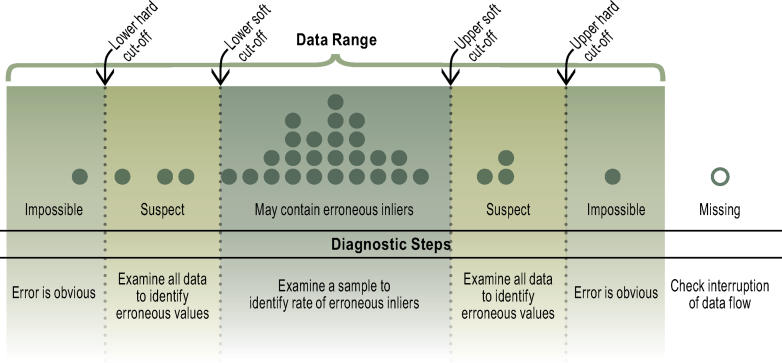
Areas within the Range of a Continuous Variable Defined by Hard and Soft Cutoffs for Error Screening and Diagnosis, with Recommended Diagnostic Steps for Data Points Falling in Each Area (Illustration: Giovanni Maki)

One procedure is to go to previous stages of the data flow to see whether a value is consistently the same. This requires access to well-archived and documented data with justifications for any changes made at any stage. A second procedure is to look for information that could confirm the true extreme status of an outlying data point. For example, a very low score for weight-for-age (e.g., −6 *Z*-scores) might be due to errors in the measurement of age or weight, or the subject may be extremely malnourished, in which case other nutritional variables should also have extremely low values. Individual patients' reports with accumulated information on related measurements are helpful for this purpose. This type of procedure requires insight into the coherence of variables in a biological or statistical sense. Again, such insight is usually available before the study and can be used to plan and program data cleaning. A third procedure is to collect additional information, e.g., question the interviewer/measurer about what may have happened and, if possible, repeat the measurement. Such procedures can only happen if data cleaning starts soon after data collection, and sometimes remeasuring is only valuable very shortly after the initial measurement. In longitudinal studies, variables are often measured at specific ages or follow-up times. With such designs, the possibility of remeasuring or obtaining measurements for missing data will often be limited to predefined allowable intervals around the target times. Such intervals can be set wider if the analysis foresees using age or follow-up time as a continuous variable.

Finding an acceptable value does not always depend on measuring or remeasuring. For some input errors, the correct value is immediately obvious, e.g., if values of infant length are noted under head circumference and vice versa. This example again illustrates the usefulness of the investigator's subject-matter knowledge in the diagnostic phase. Substitute code values for missing data should be corrected before analysis.

During the diagnostic phase, one may have to reconsider prior expectations and/or review quality assurance procedures. The diagnostic phase is labor intensive and the budgetary, logistical, and personnel requirements are typically underestimated or even neglected at the study design stage. How much effort must be spent? Cost-effectiveness studies are needed to answer this question. Costs may be lower if the data-cleaning process is planned and starts early in data collection. Automated query generation and automated comparison of successive datasets can be used to lower costs and speed up the necessary steps.

## Treatment Phase

After identification of errors, missing values, and true (extreme or normal) values, the researcher must decide what to do with problematic observations. The options are limited to correcting, deleting, or leaving unchanged. There are some general rules for which option to choose. Impossible values are never left unchanged, but should be corrected if a correct value can be found, otherwise they should be deleted. For biological continuous variables, some within-subject variation and small measurement variation is present in every measurement. If a remeasurement is done very rapidly after the initial one and the two values are close enough to be explained by these small variations alone, accuracy may be enhanced by taking the average of both as the final value.

What should be done with true extreme values and with values that are still suspect after the diagnostic phase? The investigator may wish to further examine the influence of such data points, individually and as a group, on analysis results before deciding whether or not to leave the data unchanged. Statistical methods exist to help evaluate the influence of such data points on regression parameters. Some authors have recommended that true extreme values should always stay in the analysis [25]. In practice, many exceptions are made to that rule. The investigator may not want to consider the effect of true extreme values if they result from an unanticipated extraneous process. This becomes an a posteriori exclusion criterion and the data points should be reported as “excluded from analysis”. Alternatively, it may be that the protocol-prescribed exclusion criteria were inadvertently not applied in some cases [26].

Data cleaning often leads to insight into the nature and severity of error-generating processes. The researcher can then give methodological feedback to operational staff to improve study validity and precision of outcomes. It may be necessary to amend the study protocol, regarding design, timing, observer training, data collection, and quality control procedures. In extreme cases, it may be necessary to restart the study. Programming of data capture, data transformations, and data extractions may need revision, and the analysis strategy should be adapted to include robust estimation or to do separate analyses with and without remaining outliers and/or with and without imputation.

## Data Cleaning as a Study- Specific Process

The sensitivity of the chosen statistical analysis method to outlying and missing values can have consequences in terms of the amount of effort the investigator wants to invest to detect and remeasure. It also influences decisions about what to do with remaining outliers (leave unchanged, eliminate, or weight during analysis) and with missing data (impute or not) [27–31]. Study objectives codetermine the required precision of the outcome measures, the error rate that is acceptable, and, therefore, the necessary investment in data cleaning.

Longitudinal studies necessitate checking the temporal consistency of data. Plots of serial individual data such as growth data or repeated measurements of categorical variables often show a recognizable pattern from which a discordant data point clearly stands out. In clinical trials, there may be concerns about investigator bias resulting from the close data inspections that occur during cleaning, so that examination by an independent expert may be needed.

In small studies, a single outlier will have a greater distorting effect on the results. Some screening methods such as examination of data tables will be more effective, whereas others, such as statistical outlier detection, may become less valid with smaller samples. The volume of data will be smaller; hence, the diagnostic phase can be cheaper and the whole procedure more complete. Smaller studies usually involve fewer people, and the steps in the data flow may be fewer and more straightforward, allowing fewer opportunities for errors.

In intervention studies with interim evaluations of safety or efficacy, it is of particular importance to have reliable data available before the evaluations take place. There is a need to initiate and maintain an effective data-cleaning process from the start of the study.

## Documentation and Reporting

Good practice guidelines for data management require transparency and proper documentation of all procedures [1–4,30]. Data cleaning, as an essential aspect of quality assurance and a determinant of study validity, should not be an exception. We suggest including a data-cleaning plan in study protocols. This plan should include budget and personnel requirements, prior expectations used to screen suspect data, screening tools, diagnostic procedures used to discern errors from true values, and the decision rules that will be applied in the editing phase. Proper documentation should exist for each data point, including differential flagging of types of suspected features, diagnostic information, and information on type of editing, dates, and personnel involved.

In large studies, data-monitoring and safety committees should receive detailed reports on data cleaning, and procedural feedbacks on study design and conduct should be submitted to a study's steering and ethics committees. Guidelines on statistical reporting of errors and their effect on outcomes in large surveys have been published [31]. We recommend that medical scientific reports include data-cleaning methods. These methods should include error types and rates, at least for the primary outcome variables, with the associated deletion and correction rates, justification for imputations, and differences in outcome with and without remaining outliers [25].
